# Association of annual hospital septic shock case volume and hospital mortality

**DOI:** 10.1186/s13054-022-04035-8

**Published:** 2022-06-04

**Authors:** Yan Chen, Xu-dong Ma, Xiao-hui Kang, Si-fa Gao, Jin-min Peng, Shan Li, Da-wei Liu, Xiang Zhou, Li Weng, Bin Du, China National Critical Care Quality Control Center Group

**Affiliations:** 1 Medical Intensive Care Unit, State Key Laboratory of Complex Severe and Rare Diseases, Peking Union Medical College Hospital, Peking Union Medical College and Chinese Academy of Medical Sciences, Beijing, 100730 China; 2 Department of Medical Administration, National Health Commission of the People’s Republic of China, Beijing, 100044 China; 3 Department of Critical Care Medicine, Department of Information Center, Peking Union Medical College Hospital, Peking Union Medical College and Chinese Academy of Medical Sciences, Beijing, 100730 China

**Keywords:** Septic shock, Case volume, Hospital mortality

## Abstract

**Background:**

The burden of sepsis remains high in China. The relationship between case volume and hospital mortality among patients with septic shock, the most severe complication of sepsis, is unknown in China.

**Methods:**

In this retrospective cohort study, we analyzed surveillance data from a national quality improvement program in intensive care units (ICUs) in China in 2020. Association between septic shock case volume and hospital mortality was analyzed using multivariate linear regression and restricted cubic splines.

**Results:**

We enrolled a total of 134,046 septic shock cases in ICUs from 1902 hospitals in China during 2020. In this septic shock cohort, the median septic shock volume per hospital was 33 cases (interquartile range 14–76 cases), 41.4% were female, and more than half of the patients were over 61 years old, with average hospital mortality of 21.2%. An increase in case volume was associated with improved survival among septic shock cases. In the linear regression model, the highest quartile of septic shock volume was associated with lower hospital mortality compared with the lowest quartile (*β* − 0.86; 95% CI − 0.98, − 0.74; *p* < 0.001). Similar differences were found in hospitals of respective geographic locations and hospital levels. With case volume modeled as a continuous variable in a restricted cubic spline, a lower volume threshold of 40 cases before which a substantial reduction of the hospital mortality rate was observed.

**Conclusions:**

The findings suggest that hospitals with higher septic shock case volume have lower hospital mortality in China. Further research is needed to explain the mechanism of this volume–outcome relationship.

**Supplementary Information:**

The online version contains supplementary material available at 10.1186/s13054-022-04035-8.

## Introduction

Sepsis, as a leading cause of death, is estimated to account for 11 million deaths in 2017 worldwide [1]. Our previous study suggests that the sepsis-related mortality rate was 66.7 (95% confidence interval [CI] 66.4–67.0) deaths per 100,000 population in China [2]. Strategies in the management of sepsis are urgently needed to reduce the sepsis burden.

Timely sepsis treatment such as timely administration of antibiotics and early resuscitation is of proven effectiveness in sepsis [3]. Consequently, there have been ongoing efforts to establish timely sepsis treatment protocols such as 3-h and 6-h bundles to improve survival [4]. Nevertheless, the overall hospital mortality rate of sepsis remains as high as 26.7% [5]. In other critical conditions requiring time-sensitive treatment including trauma [6] and acute myocardial infarction [7], patients are more likely to survive when treated in the high-volume centers which may lead to greater experience and medical resources. Given the distribution of quality and accessible medical resources at China hospitals [8], it is likely that disparities may exist in the care and outcomes for patients with sepsis, and septic shock, the most severe complication of sepsis in China [9].

A previous study in the USA showed improved outcomes in high-volume hospitals among patients with septic shock [10]. In contrast, the other two studies in the US [11] and the UK [12] did not show an association between hospital volume and septic shock-related mortality. However, those studies were limited to retrospective International Classification of Disease (ICD) code-based identification of sepsis and septic shock [10, 11], high-income countries (HICs) [10–12] and non-specific organ dysfunction [10–12].

Our study aimed to examine the relationship between annual septic shock case volume and hospital mortality in China. We used data from the national quality improvement program in China to explore the relationship between in-hospital mortality rates and case volume of septic shock.

## Method

### Study design and data sources

The study protocol was approved by the institutional review board of Peking Union Medical College Hospital; the approval included a waiver for the informed consent of the patients and physicians.

We conducted a nationwide retrospective cohort study using the surveillance data from the national quality improvement (QI) program in ICUs in China. The program was designed and led by China National Critical Care Quality Control Center (China-NCCQC), the official national department regulating ICU quality control in China [13]. The national ICU QI program is a hospital-based continuous QI initiative, with multifaceted intervention implemented in multicenter voluntarily. The relevant de-identified data regarding the quality control indicators of hospitals and ICUs were prospectively collected from each hospital. The data were collected and monitored annually through the database of the National Clinical Improvement System (https://ncisdc.medidata.cn/login.jsp) since 2016 when the QI program was initiated.

All public secondary and tertiary hospitals have access to the program. Tertiary hospital proportion (%) of total hospitals in different provinces and cities is shown in Additional file 1: Fig. S1. The criteria of the enrollment for the ICUs: (1) are more than five beds; (2) are capable of performing quality control programs in hospital-acquired infections such as catheter-related bloodstream infection (CRBSI), ventilator-associated pneumonia (VAP), and deep vein thrombosis (DVT); (3) meet the requirements for construction and management of ICUs in China [14–16]. Hospital characteristics such as geographic location, type and level of the hospitals, and hospital volume were collected.

Fifteen ICU quality control indicators are adopted in the program, based on the recommendations by the National Health Commission of the People’s Republic of China on April 10, 2015 [17]. The indicators were described as previously [13], and listed as follows: (1) three structural indicators including proportion of ICU patients out of all total inpatients, proportion of ICU bed occupancy out of the total inpatient bed occupancy, proportion of ICU patients with acute physiology, and chronic health evaluation (APACHE) II scores ≥ 15 out of all ICU patients; (2) four process indicators including 3-h Surviving Sepsis Campaign (SSC) bundle compliance rate, 6-h SSC bundle compliance rate, microbiology specimen collection before antibiotic therapy, DVT prophylaxis rate); (3) eight outcome indicators including unplanned endotracheal extubation rate, reintubation rate within 48 h, rate of unplanned transfer to ICU, ICU readmission rate within 48 h, VAP incidence rate, CRBSI incidence rate, catheter-associated urinary tract infection incidence rate, ICU mortality.

Three of fifteen indicators mentioned above were related to septic shock, including 3-h, 6-h SSC bundle compliance rate and microbiology specimen collection before antibiotic therapy. More quality indicators related to septic shock were updated in 2019. The Septic Shock 3.0 definition was used to define septic shock in the program [18]. Septic shock was defined as life-threatening organ dysfunction due to dysregulated host response caused by infection, SOFA ≥ 2 and lactate ≥ 2 mmol/L while requiring vasoactive drug support to maintain blood pressure after fluid resuscitation. Septic shock-related indicators include demographic characteristics, infection site, pathogens, antibiotics used, and hospital mortality rate of septic shock.

We collected information from 1902 hospitals across China from January 2020 to December 2020, when more quality indicators related to septic shock have been updated. Data regarding hospital and ICU characteristics, septic shock-related quality control indicators, such as the number of hospital beds and the rate of 3-h SSC bundle compliance was included. Hospitals, instead of individual patients, were the unit of analysis.

### Variables and risk adjustment

The exposure variable was annualized hospital volume, defined as the number of cases of septic shock during the 1-year study period. The primary outcome was hospital mortality of septic shock cases. The relationship between septic shock case volume and hospital mortality was determined with volume as a continuous variable and after categorizing volume into quartiles. The reference categories are the lowest-volume hospital and the lowest-volume quartile, respectively. The rate of hospital mortality was natural log-transformed in the analyses because it was not normally distributed.

Risk-adjustment variables include the type of hospitals, geographic location, adherence to 3-h SSC bundle, site of infection (sites of the bloodstream, urinary, skin, central nervous system, lung, bone, abdominal, gastrointestinal), infection management, infection training, infection monitor, infection contingency plan, infection performance, microbiology specimen collection before antibiotic therapy, the proportion of APACHE II score more than 15, and proportion of age more than 61 years old. 6-h SSC bundle compliance was not incorporated as a confounding variable for the possible collinearity with 3-h SSC bundle compliance. The infection site of the biliary was also not used in the model for the potential collinearity with the infection site of the lung.

### Statistical analyses

Continuous data were compared with analysis of variance and non-continuous dichotomous data was compared by the chi-square test across the quartiles of septic shock case volume. We evaluated correlations of rates of each paired combination of the sites of infections, 3-h SSC bundle compliance, 6-h SSC bundle compliance, sex, and age, using Spearman rank coefficients and visualized the relationships with heatmap. The correlation between 3-h SSC bundle compliance and 6-h SSC bundle compliance was also visualized using scatter plots with a linear regression line with 95% CI.

Multilevel linear modeling was performed to determine associations between quartiles of septic shock case volume and hospital mortality of septic shock. A stepwise modeling method was used to estimate the effectiveness of additional groups of covariates on the (adjusted) volume–outcome relationship. Interaction *p* values comparisons between case volume quartiles were made using the Turkey's test. Additional adjustments were made for the type of hospitals, geographic location, site of infection (sites of the bloodstream, urinary, skin, central nervous system, lung, bone, abdominal, gastrointestinal), the proportion of APACHE II score ≥ 15, and proportion of age ≥ 60 (model 1); microbiology specimen collection before antibiotic therapy (model 2); infection management, infection training, infection monitor, infection contingency plan, infection performance (model 3); adherence to 3-h bundle (model 4). A *p* value less than 0.05 was considered significant. Moreover, we performed a series of analyses stratified by geographic location (east, middle, west, northeast), and hospital-level (secondary and tertiary) in the fully adjusted model (model 4) with limitations to the respective hospitals.

We also used the restricted cubic splines with five knots at the 5th, 35th, 50th, 65th, and 95th centiles for the assessment of volume as a continuous variable. The model was adjusted for all potential confounding variables.

All statistical analyses were performed using R (version 4.0.0, R studio, Boston, MA).

## Results

### Hospital and septic shock case characteristics

During the study period, a total of 134,046 septic shock cases across 1902 hospitals in China were enrolled. Characteristics of the hospitals by quartile of septic shock case volume are shown in Table 1. Compared with lowest case volume quartile hospitals, the highest quartile of septic shock case volume hospitals were more likely public hospitals, tertiary hospitals. Higher case volume quartile hospitals had higher healthcare facility volume, higher microbiology specimen collection before antibiotic therapy, and better performance of protocolized infection control, compared with lower quartile hospitals. Hospitals at higher septic shock case volume were more likely to implement 3-h and 6-h SSC bundles compared with the lowest quartile hospitals. In this septic shock cohort, the median septic shock volume per hospital was 33 cases (interquartile range 14–76 cases), 41.4% were female, and more than half of the patients were over 61 years old, with average hospital mortality of 21.2%. The top three most common infection sites among septic shock cases were lung, bloodstream, and urinary tract.

**Table 1:** Characteristics of septic shock cases and enrolled hospitals and intensive care units

	Septic shock case volume quartile				*p* value^a^
	First quartile, 1–13 cases per year	Second quartile, 14–32 cases per year	Third quartile, 33–75 cases per year	Fourth quartile, > 75 cases per year	
*Hospitals and ICUs*					
Number of hospitals, *n*	475	468	478	481	
Geographic location, *n* (%)					0.763
East	193 (40)	179 (38)	191 (39)	204 (42)	
Middle	102 (21)	118 (25)	104 (21)	114 (23)	
West	158 (33)	150 (32)	162 (33)	136 (28)	
Northeast	22 (4)	21 (4)	21 (4)	27 (5)	
Type of hospitals, *n* (%)					0.001
Public	432 (91)	430 (92)	440 (92)	466 (97)	
Private	43 (9)	38 (8)	38 (8)	15 (3)	
Level of hospitals, *n* (%)					< 0.0001
Tertiary	139 (29)	226 (48)	277 (57)	388 (80)	
Secondary	331 (69)	238 (50)	199 (41)	91 (18)	
Healthcare facility volume, *n*					
Hospital volume per year	18,616 ± 16,069	26,460 ± 27,795	34,297 ± 28,557	60,618 ± 55,195	< 0.0001
ICU volume per year	414 ± 1509	597 ± 2337	641 ± 586	1463 ± 2119	< 0.0001
Hospital bed days/1000 patients	153 ± 102	224 ± 211	331 ± 1036	522 ± 1144	< 0.001
ICU bed days/1000 patients	3 ± 13	3 ± 4	6 ± 33	11 ± 37	< 0.0001
Guideline compliance, %					
3-h Surviving Sepsis Campaign bundle	73 ± 32	79 ± 25	78 ± 25	78 ± 24	0.004
6-h Surviving Sepsis Campaign bundle	71 ± 33	77 ± 27	77 ± 27	76 ± 24	0.002
Microbiology specimen collection before antibiotic therapy, %	68 ± 34	75 ± 27	77 ± 29	80 ± 24	< 0.001
Protocolized Infection control, *n* (%)					
Infection management	434 (91)	448 (96)	466 (97)	467 (97)	< 0.001
Infection training	433 (91)	433 (93)	459 (96)	467 (97)	< 0.001
Infection monitor	435 (92)	439 (94)	466 (97)	472 (98)	< 0.001
Infection contingency plan	443 (93)	445 (95)	467 (98)	471 (98)	< 0.001
Infection performance	318 (67)	325 (70)	363 (76)	350 (73)	0.015
ICU mortality rate, %	11 ± 10	12 ± 10	12 ± 10	12 ± 9	0.530
*Septic shock cases*					
Female, %	45 ± 20	40 ± 11	40 ± 10	41 ± 9	< 0.0001
Age group, %	0 ± 0	0 ± 0	0 ± 0	0 ± 0	
Less than 18 years old	3 ± 15	2 ± 10	3 ± 13	2 ± 8	0.263
18–30 years old	3 ± 13	2 ± 4	3 ± 4	3 ± 4	0.027
31–40 years old	4 ± 11	4 ± 6	5 ± 6	6 ± 5	< 0.0001
41–50 years old	8 ± 14	9 ± 9	10 ± 8	10 ± 6	< 0.0001
51–60 years old	15 ± 18	16 ± 11	17 ± 10	17 ± 8	0.070
61–70 years old	25 ± 22	25 ± 13	25 ± 11	23 ± 9	0.062
71–80 years old	26 ± 23	24 ± 13	23 ± 12	23 ± 11	0.016
More than 80 years old	18 ± 22	18 ± 15	16 ± 14	15 ± 11	0.026
Site of infection, %					
Bloodstream	16 ± 26	17 ± 21	16 ± 20	15 ± 15	0.850
Catheter related bloodstream	4 ± 12	5 ± 10	3 ± 7	3 ± 5	0.043
Lung	44 ± 31	42 ± 22	41 ± 23	41 ± 22	0.294
Abdomen	18 ± 22	17 ± 14	18 ± 14	16 ± 11	0.113
Urinary tract	14 ± 20	12 ± 11	13 ± 11	12 ± 9	0.378
Central nervous system	2 ± 9	2 ± 4	2 ± 3	3 ± 4	0.043
Biliary tract	7 ± 14	7 ± 7	7 ± 6	7 ± 6	0.720
Gastrointestinal tract	5 ± 11	6 ± 8	7 ± 9	6 ± 7	0.018
Bone	0 ± 5	0 ± 1	0 ± 1	0 ± 0	0.205
Skin and soft tissue	3 ± 11	3 ± 5	3 ± 4	3 ± 3	0.196
Others	2 ± 10	2 ± 6	2 ± 4	3 ± 6	0.177
Proportion of APACHE II score ≥ 15, %	57 ± 27	61 ± 24	65 ± 23	62 ± 24	< 0.0001
Hospital mortality of septic shock cases, %	24 ± 26	24 ± 20	20 ± 17	18 ± 15	< 0.0001

Data presented as mean ± SD, *n* (%). *p* value represents comparisons between all quartiles

Hospital bed days/1000 patients, days of hospital bed occupancy per 1000 patients

ICU bed days/1000 patients, days of ICU bed occupancy per 1000 patients

*APACHE* Acute physiology and chronic health evaluation, *ICU* intensive care unit

^a^Comparisons between groups were made using the ANOVA test for continuous variables and the Chi-Squared test for categorical variables

### Correlations between variables

**Fig. 1:**
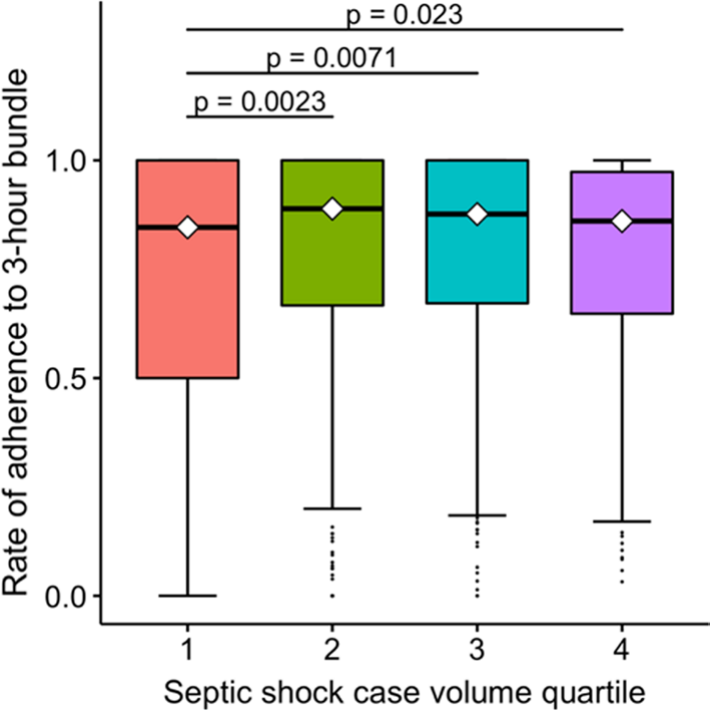
Box plot of hospital-level rate of adherence to 3-h bundle showing median, interquartile range, range (with outliers), and median (diamonds). All quartiles compared to the lowest quartile (quartile 1 as reference)

Rates of 3-h SSC bundle compliance strongly correlated with 6-h bundle compliance (Spearman rank correlation test, *ρ* = 0.7; *p* < 0.001) (Additional file 1: Figs. S2 and S3). Median rates of adherence to the 3-h SSC bundle of each septic case volume quartile (second, third, and fourth quartiles) were significantly higher than the lowest quartile (first quartile) (Fig. 1).

### Association of septic shock case volume with hospital mortality

In the linear model, the highest quartile of septic shock case volume was associated with lower hospital mortality of septic shock compared with the lowest quartile (*β* − 0.86; 95% CI − 0.98, − 0.74; *p* < 0.001). After controlling for additional confounding variables in all four adjusted models, including the fully adjusted model 4, patients in quartile 2, 3, and 4 had lower mortality than its immediate lower quartile (Q2 vs. Q1, *β* − 0.29, *p* < 0.001; Q3 vs. Q2, *β* − 0.33, *p* < 0.001; Q4 vs. Q3, *β* − 0.17, *p* = 0.037) (Table 2). As shown in Additional file 1: Table S1, we performed a fully adjusted analysis to address whether the volume–outcome relationship identified existed in different geographic locations and hospital levels. The substantial inverse volume-mortality relationship among septic shock patients was observed in all geographic locations and all types of hospitals.

**Table 2:** Association between septic shock case volume quartile and hospital mortality of septic shock

Sample	Unadjusted	Model 1	Model 2	Model 3	Model 4	Interaction *p* values^a^
First quartile	Ref	Ref	Ref	Ref	Ref	–
Second quartile	− 0.35 (− 0.47, − 0.22)	− 0.30 (− 0.44, − 0.16)	− 0.29 (− 0.43, − 0.14)	− 0.28 (− 0.43, − 0.13)	− 0.29 (− 0.43, − 0.14)	< 0.001^b^
Third quartile	− 0.69 (− 0.81, − 0.56)	− 0.63 (− 0.78, − 0.49)	− 0.62 (− 0.77, − 0.47)	− 0.62 (− 0.77, − 0.47)	− 0.62 (− 0.77, − 0.47)	< 0.001^c^
Fourth Quartile	− 0.86 (− 0.98, − 0.74)	− 0.80 (− 0.95, − 0.66)	− 0.79 (− 0.95, − 0.64)	− 0.79 (− 0.94, − 0.64)	− 0.79 (− 0.94, − 0.64)	0.037^d^

Model 1, model adjusted for type of hospitals, geographic location, site of infection (sites of bloodstream, urinary, skin, central nervous system, lung, bone, abdominal, gastrointestinal), the proportion of APACHE II score ≥ 15, and proportion of age ≥ 60

Model 2, adjusted for model 1 + microbiology specimen collection before antibiotic therapy

Model 3, adjusted for model 2 + infection management, infection training, infection monitor, infection contingency plan, infection performance

Model 4, adjusted for model 3 + adherence to 3-h bundle

^a^Interaction *p* values comparisons of Model 4 between case volume quartiles were made using the Turkey's test

^b^Comparison between second quartile and first quartile

^c^Comparison between third quartile and second quartile

^d^Comparison between fourth quartile and third quartile

**Fig. 2:**
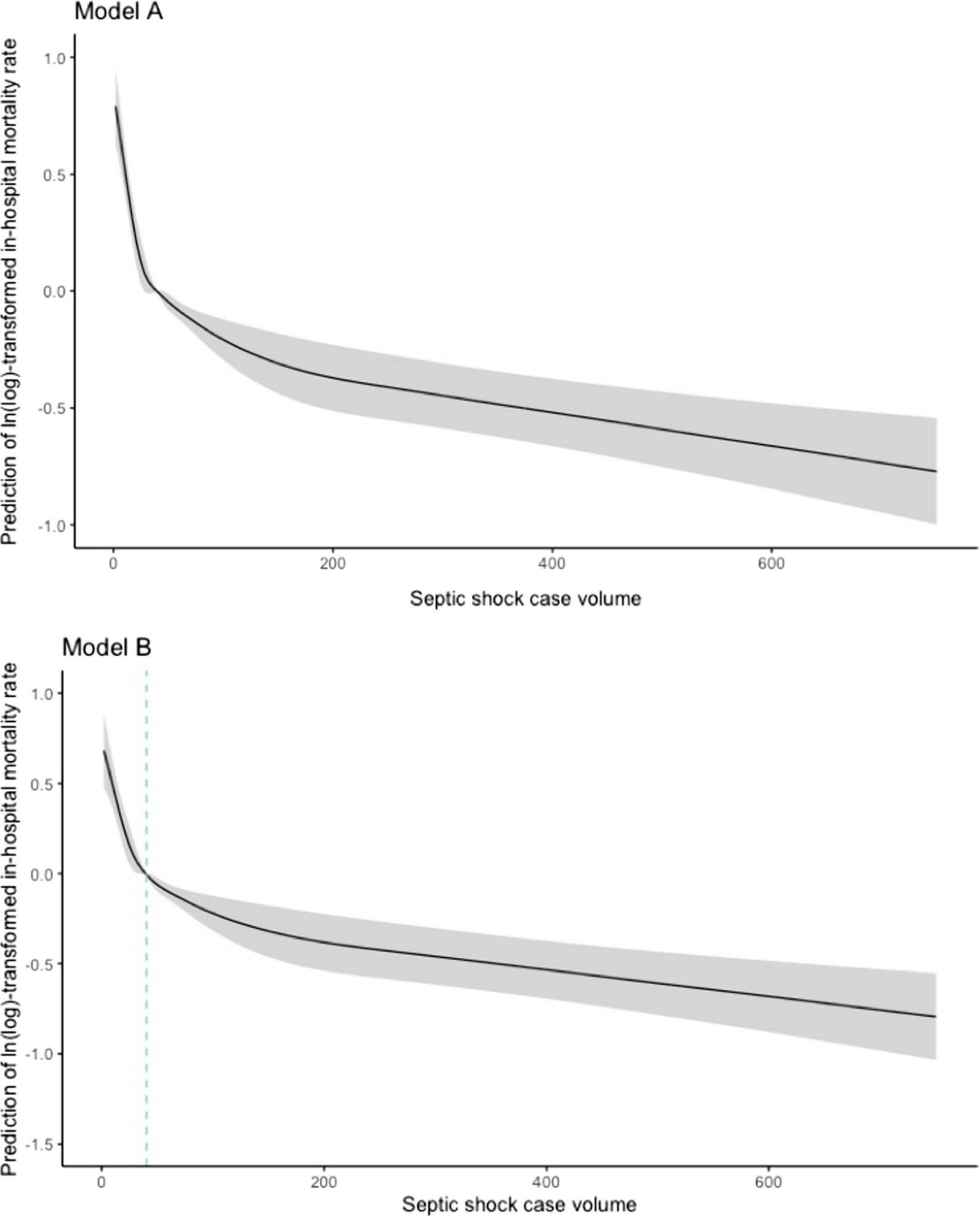
Association of septic shock case volume and the ln(log)-transformed in-hospital mortality rate. Predictions of ln(log)-transformed in-hospital mortality rate are indicated by solid lines and 95% CI by shaded areas; dashed vertical line indicates the threshold before which an increase in volume resulted in a substantial reduction in estimated mortality. Knots were placed at 5th, 35th, 50th, 65th, and 95th centiles of septic shock case volume distribution. **Model A** was unadjusted (*R*^2^ 0.141, *p* for nonlinearity < 0.0001). **Model B** was adjusted for type of hospitals, geographic location, adherence to 3-h SSC bundle, site of infection (sites of bloodstream, urinary, skin, central nervous system, lung, bone, abdominal, gastrointestinal), microbiology specimen collection before antibiotic therapy, infection management, infection training, infection monitor, infection contingency plan, infection performance, and the proportion of APACHE II score ≥ 15 and proportion of age ≥ 60 (*R*^2^ 0.17, *p* for nonlinearity < 0.0001)

In Fig. 2, we used restricted cubic splines to flexibly model and visualize the relation of case volume as a continuous variable and septic shock mortality. The plot showed a substantial reduction of the risk of mortality within the lower range of septic shock case volume (below case volume of around 40) and then turned relatively flat (*R*^2^ 0.17, *p* for nonlinearity < 0.0001).

## Discussion

Our study confirmed the relationship between higher case volume and lower risk hospital mortality among septic shock cases. The effect was consistent in the linear model and restricted cubic splines. The effect was robust after controlling for relevant confounders and after a sensitivity analysis in which various stratification of hospitals and modeling assumptions were examined. The study also found a lower septic shock volume threshold of 40 cases, before which a substantial reduction of the hospital mortality rate was observed.

Our results were consistent with the subgroup analysis of a previous study with a cohort of nearly 410,000 septic shock patients from hospitals across the USA [10]. Another study in the US failed to find the volume–outcome association among patients with septic shock [11]. The explanation might be the limited hospital representatives. The Premier enhanced administrative database was used in the latter study, and it included hospitals that were predominantly small-to-mid-size nonteaching facilities [19]. Instead of the ICD-9 code with claim data in both the US studies, the septic shock 3.0 definition with data prospectively collected was used to identify septic shock in our study. Accordingly, patients with septic shock are more likely correctly coded [20].

A subgroup analysis of ICU patients in the UK demonstrated a similar negative trend toward a volume effect in septic shock patients, though no significant difference was found (*β* [SE], − 0.00051 [0.00026]; *p* = 0.052) [12]. The mean APACHEII score was 18.4 in the UK study, which is comparable to our study (over half of the cases had APACHE II score ≥ 15). The study used the Case Mix Programme Database for data collection, which identified cases according to the reason for admission to ICU. As a result, cases of nosocomial sepsis and septic shock might have been missed. Meanwhile, there is a high incidence and mortality among these patients. According to a meta-analysis, the pooled proportion of septic shock cases with a hospital origin was 35.8% [21]. Rodriguez F and his colleagues showed higher 28-day mortality of hospital-acquired septic shock than community-acquired (52% vs. 42%) [22]. Therefore, the statistical nonsignificant volume–outcome relationship of the UK study may be attributed to the omission of more lethal nosocomial cases that required higher ICU support.

Studies regarding volume–outcome relationship among patients with sepsis and septic shock were all conducted in HICs. However, disparities in resources and care between HICs and the rest of the world limit generalization of these findings to China, one of the most populous countries. Our result showed a significant association between septic shock case volume and hospital mortality in China, adding new knowledge on volume–outcome association in countries other than HICs.

The UK study mentioned above reported a spline shape with a relatively mild descent curve until volume threshold around 200 patients, which is different from our steep descent curve before septic shock volume of 40 cases. This might attribute to the different medical resource distribution between UK and China. Over the recent decades, great efforts and progress have been made to improve equal access to care in China. The healthcare funding has been quadrupled since 2009, when the launch of the healthcare reform [23]. However, gaps remain in quality of care, which matches our result. The substantial reduction of mortality with the increasing volume in the low range volume (over half of all cases) suggests an urgent need for medical quality improvement. Especially the hospitals with annual septic shock case volume lower than 40 cases from countries other than HICs. Accordingly, the median annual ICU admission was 401 (IQR 234-711) in low-income countries, the ICU capacity of which is similar to the first quartile in our study [24].

The possible reasons for the volume–outcome relationship might include the implementation of best practices, such as multidisciplinary teams, and protocols for sepsis treatment. Several studies demonstrated a survival benefit with evidence-based processes of care [25–27]. Moreover, sepsis protocol use enforced as a strategy in New York State also showed a reduction rate of death in hospitals with protocolized sepsis care among patients with sepsis [28]. The previous study with limited indicators (lactate measurement, norepinephrine as the first vasopressor, and avoiding starch products) failed to find mediation effect of sepsis process care on volume–outcome association. Our study showed that hospitals in the highest volume category were more likely to implement a 3-h SSC bundle than the lowest volume category. However, the volume–outcome relationship that we observed was independent of protocol used by a 3-h SSC bundle. Our findings suggest that hospital volume is another important determinant of outcome among septic shock patients, and the regional centers for septic shock might benefit the patients.

Besides protocol use, further information is needed to identify the best way to improve outcomes. A feasible alternative method might be the regionalization of critical care, which allows more access to large regional care centers as a study indicated in several surgery conditions [29]. However, there are quantity inequities in the geographical and hospital distribution of healthcare resources in China [30], different geographic locations, and types of hospitals may have a mediation on the volume–outcome relationship. Our study showed improved adjusted outcomes at highest volume hospitals than lowest volume hospitals in respective geographic location and hospital level.

Our study has strengths. We used the prospectively collected surveillance data from ICUs' quality improvement program across China, which has broad coverage and representativeness of countries other than HICs. By including different hospital levels from 34 provinces of China, the study evaluated the relationship between case volume and hospital mortality within the entire adult septic shock population.

Our study has limitations. Firstly, the data we used lack individual-level information. As a result, the adjustment of detail risk such as organ dysfunction was limited. Secondly, the septic shock cases account for a small proportion of ICU admissions in the first quartile (13/414, 3%) which seems unrepresentative. This is due to the inclusion of various types of ICUs such as cardiac intensive care unit (CICU) and neurological ICU in the nationwide quality improvement program. However, this should be valuable in specific ICUs that septic shock is not a common reason for admission. For example, according to a clinical investigation of shock epidemiology of Mayo Clinic CICU between 2007 and 2018, the common reason for admission was cardiac disease, while septic shock comprised only 1.5% (194/13222) of admissions [31]. Thirdly, the stratifications by different ICU departments (e.g., medical, surgical ICUs) are unavailable. Lastly, “ICU bed-day” instead of “ICU beds” was used to measure the ICU capacity in our study. The dynamically changing ICU beds may not be a suitable quality indicator for healthcare institutions under continued development in China. Despite these limitations, the volume–outcome relationship is well elucidated with the use of hospital-level data in our study and the result is representative of different types of hospitals across China.

## Conclusion

Our results suggest that hospitals with a high septic shock case volume were associated with reduced hospital mortality in China, and a volume threshold associated with an obvious reduction in mortality was identified. Further research is needed to explain the mechanism of this volume–outcome relationship.

## Supplementary Information

**Additional file 1. Fig. S1.** Tertiary hospital proportion (%) of all types of hospitals in different provinces and cities. **Fig. S2.** Correlation of 3-hour bundle and 6-hour bundle compliance with linear regression with 95% CI. Spearman rank correlation test, *p* = 0.7; *p* < 0.001. **Fig. S3.** Correlation between sites of infection, age, sex, and proportion of APACHE II score more than 15. Abbreviations: CNS, central nervous system; older, more than 60 years old. **Table S1** Association Between Septic shock case volume quartile and hospital mortality of septic shock.

## Data Availability

The datasets analyzed during the current study are available from the corresponding author on reasonable request.
