# Supplementary material for: Association of annual hospital septic shock case volume and hospital mortality
Source: Crit Care. 2022 Jun 4;26:161. doi: 10.1186/s13054-022-04035-8 (PMC9166431; doi:10.1186/s13054-022-04035-8)
Supplement: Supplementary file 1 — Additional file 1. Fig. S1. Tertiary hospital proportion (%) of all types of hospitals in different provinces and cities. Fig. S2. Correlation of 3-hour bundle and 6-hour bundle compliance with linear regression with 95% CI. Spearman rank correlation test, p = 0.7; p < 0.001. Fig. S3. Correlation between sites of infection, age, sex, and proportion of APACHE II score more than 15. Abbreviations: CNS, central nervous system; older, more than 60 years old. Table S1 Association Between Septic shock case volume quartile and hospital mortality of septic shock. [file 13054_2022_4035_MOESM1_ESM.docx]

**Supplementary appendix to**

**Association of Annual Hospital Septic Shock Case Volume and Hospital Mortality**

**Definitions and explanations**

Performance of protocolized infection control included infection management, infection training, infection monitor, infection contingency plan and infection performance.

- Infection management, defined as the availability of a management team for infectious disease in a hospital.

- Infection training, defined as the availability of training system for infectious disease in a hospital.

- Infection monitor, defined as the availability of a monitoring procedure for infectious disease in a hospital.

- Infection contingency plan, defined as the availability of an emergency plan for infectious disease in a hospital.

- Infection performance, defined as the availability of performance assessment for infectious disease management in a hospital.

Fig. S1. Tertiary hospital proportion (%) of all types of hospitals in different provinces and cities


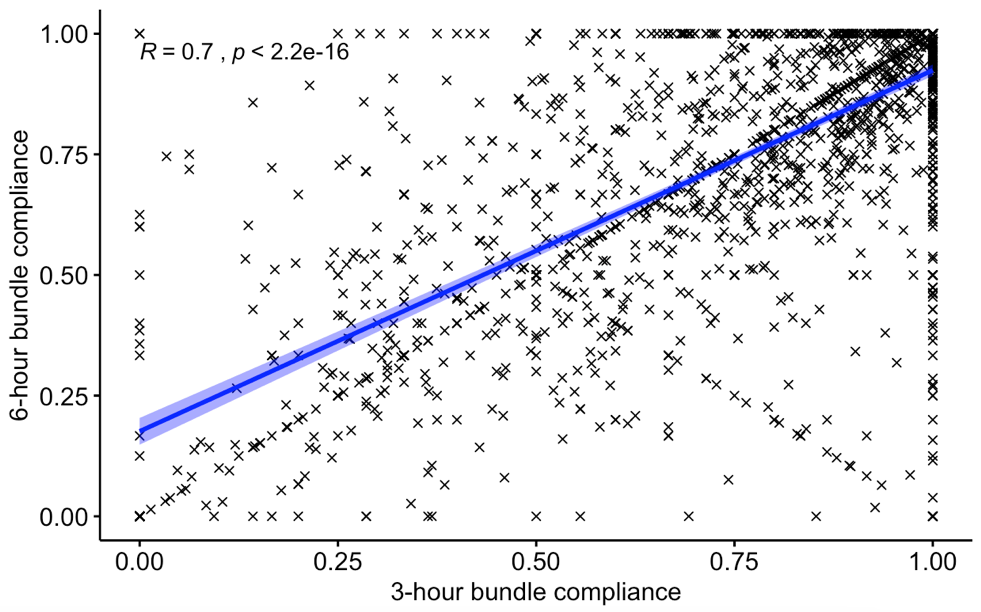


Fig.S2. Correlation of 3-hour bundle and 6-hour bundle compliance with liner regression with 95% CI.

Spearman rank correlation test, ρ = 0.7; p < 0.001.


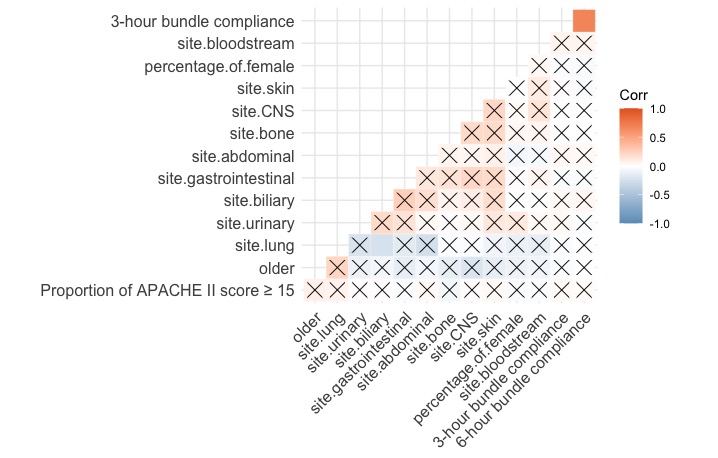


Fig.S3 Correlation between sites of infection, age, sex, and proportion of APACHE II score more than 15.

Abbreviations: CNS, central nervous system; older, more than 60 years old

Table S1 Association Between Septic shock case volume quartile and hospital mortality of septic shock

|  | **Septic Shock Case Volume Quartile** | | | |
| --- | --- | --- | --- | --- |
| **Analysis Sample** | **First Quartile,**  **1-13 cases per year**  **β* (95% CI)** | **Second Quartile,**  **14-32 cases per year**  **β* (95% CI)** | **Third Quartile,**  **33-75 cases per year**  **β* (95% CI)** | **Fourth Quartile,**  **>75 cases per year**  **β* (95% CI)** |
| Whole septic shock cohort^§^ | Ref | -0.29 (-0.43, -0.14) | -0.62( -0.77, -0.47) | -0.79(-0.94, -0.64) |
| East^¶^ | Ref | -0.27(-0.49, -0.04) | -0.63(-0.86, -0.40) | -0.80(-1.04, -0.56) |
| Northeast^¶^ | Ref | -0.78(-1.74, 0.18) | -1.18(-2.12, -0.24) | -0.95(-1.83, -0.08) |
| West^¶^ | Ref | -0.33(-0.58, -0.07) | -0.59(-0.85, -0.34) | -0.78(-1.0, -0.64) |
| Middle^¶^ | Ref | -0.24(-0.61, 0.13) | -0.60(-0.97, -0.23) | -0.72(-1.11, -0.34) |
| Secondary hospitals^†^ | Ref | -0.29(-0.47, -0.12) | -0.65(-0.84, -0.47) | -0.97(-1.2, -0.73) |
| Tertiary hospitals^†^ | Ref | -0.27(-0.52, -0.01) | -0.56(-0.81, -0.32) | -0.71(-0.95, -0.47) |

*Refers to percent differences in ln-transformed rate of hospital septic shock mortality compared with reference category, obtained from linear regression.

^§^Model adjusted for all covariates including type of hospitals, geographic location, adherence to 3-hour bundle, site of infection (sites of bloodstream, urinary, skin, central nervous system, lung, bone, abdominal, gastrointestinal), microbiology specimen collection before antibiotic therapy, infection management, infection training, infection monitor, infection contingency plan, infection performance, and the proportion of APACHE II score ≥ 15 and proportion of age ≥ 60.

^¶^ Model adjusted for all covariates expect geographic location

^†^ Model adjusted for all covariates expect type of hospital
